# araCNA: somatic copy number profiling using long-range sequence models

**DOI:** 10.1093/nargab/lqaf124

**Published:** 2025-09-09

**Authors:** Ellen Visscher, Christopher Yau

**Affiliations:** Nuffield Department for Women’s & Reproductive Health, University of Oxford, Women’s Centre, John Radcliffe Hospital, Oxford OX3 9DU, United Kingdom; Nuffield Department for Women’s & Reproductive Health, University of Oxford, Women’s Centre, John Radcliffe Hospital, Oxford OX3 9DU, United Kingdom

## Abstract

Somatic copy number alterations (CNAs) are hallmarks of cancer. Current algorithms that call CNAs from whole-genome sequenced (WGS) data have not exploited deep learning methods owing to computational scaling limitations. Here, we present a novel deep-learning approach, araCNA, trained only on simulated data that can accurately predict CNAs in real WGS cancer genomes. araCNA uses novel transformer alternatives (e.g. Mamba) to handle genomic-scale sequence lengths (∼1M) and learn long-range interactions. Results are extremely accurate on simulated data, and this zero-shot approach is on par with existing methods when applied to 50 WGS samples from the Cancer Genome Atlas. Notably, our approach requires only a tumour sample and not a matched normal sample, has fewer markers of overfitting, and performs inference in only a few minutes. araCNA demonstrates how domain knowledge can be used to simulate training sets that harness the power of modern machine learning in biological applications.

## Introduction

Somatic copy number alterations (CNAs) are genomic regions that are amplified or deleted when somatic cells replicate. They are a hallmark of many cancers and a driving factor of tumorigenesis [1], driving the amplification of oncogenes [2, 3]. CNA abundance is known to be associated with disease stage, prognosis, and response to treatment, and particular cancer types can be characterized by specific classes of structural variation, often in specific chromosomal regions [4]. Accurate CNA profiles of cancer samples are important for downstream analysis such as association studies to identify underlying cancer signatures or as a prognostic biomarker [5].

**Figure 1.**
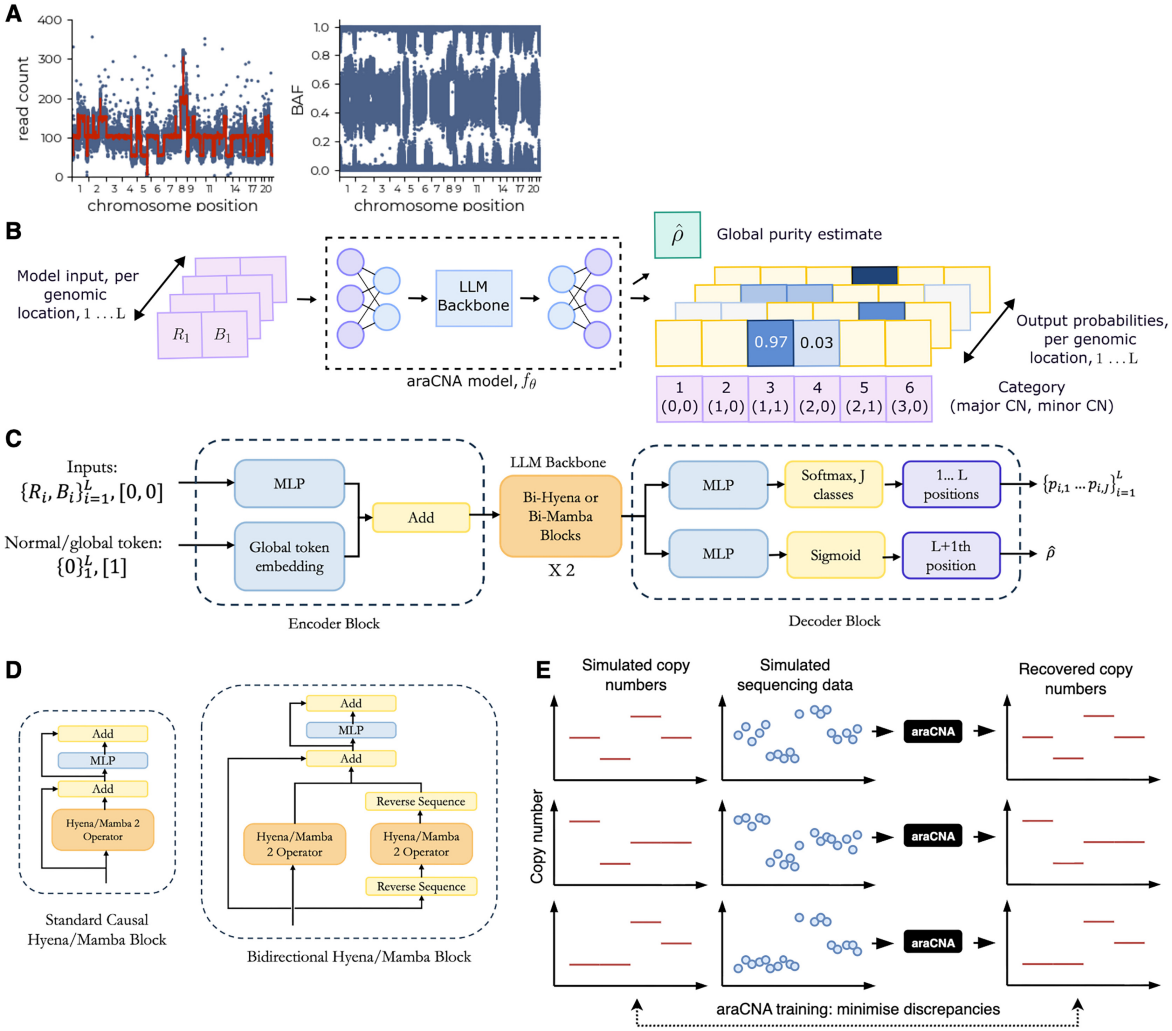
Overview of araCNA model. The (**A**) input data [read count and B allele frequency (BAF)] is (**B**) converted by araCNA into a sequence of probabilistic copy number calls and global parameter estimates. The araCNA model architecture (**C**) contains bi-directional variants (**D**) of the causal Hyena or Mamba blocks. (**E**) The model is trained on simulated data.

**Figure 2.**
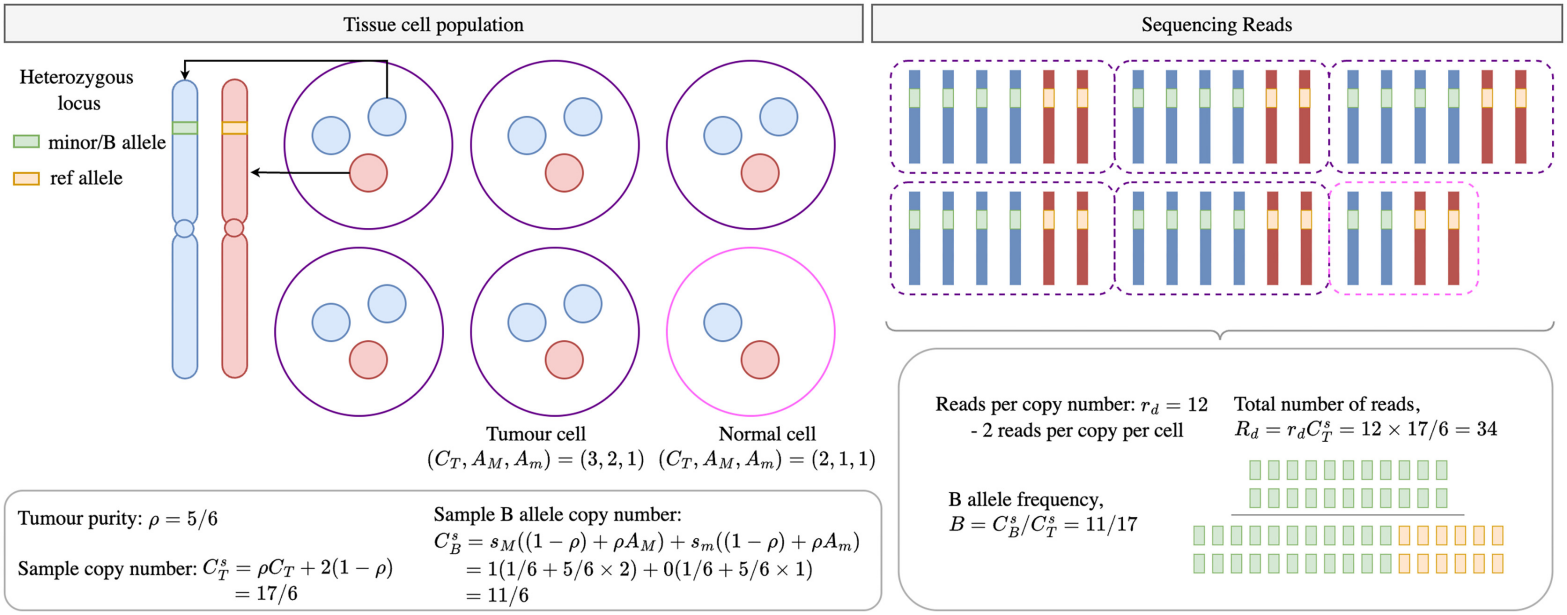
Mathematical construction of copy number calling. Illustration of how purity, copy number, read depth per copy number, and heterozygous loci result in measured read depth and BAF.

The copy number landscape of a tumour can be profiled using array- and sequencing-based technologies, where increases and decreases in signal intensity or sequence read depth correspond to gains and losses of genomic segments [6, 7]. Allelic information from single nucleotide polymorphisms (SNPs) can also be exploited to determine copy number (see Figs 1 and 2). Data can then be processed using CNA calling algorithms to derive copy number states.

CNA calling algorithms generally consist of an approach to segment the genome into regions of constant copy number and a copy number calling or classification step (into deletion, duplication, etc.). Methods adopt a variety of approaches. This includes calling total copy numbers only [8–11], while others provide allele-specific major and minor copy number calling capabilities [12, 13]. Others adopt a hybrid approach, first calling total copy numbers and then assigning these to major/minor copy numbers [14, 15]. Approaches that account for tumour heterogeneity can also provide subclonal copy numbers [16–19], again sometimes calling allele-specific copy numbers [20–23]. When multiple tumour samples are available from the same individual, information from across samples can be used to posit evolutionary trees upon which somatic point mutations and CNAs can be placed [24, 25].

Standard CNA callers adopt the use of segmentation algorithms or hidden Markov models that are effective at processing one cancer sequencing sample at a time, but the low information capacity of these methods or models means they cannot learn [26, 27]. CNA callers process every sample as though it were the very first sample they have seen. Therefore, despite large-scale mapping exercises such as the Pan-Cancer Genome Atlas studies [5], few new CNA calling approaches have been developed.

While many areas of ’omics analysis have been heavily influenced by deep learning in recent years, the technology has had relatively little impact on CNA calling algorithms. For example, the use of transformer-based models has led to the creation of massive foundation models for single-cell and integrative ’omics [28]. However, the quadratic scaling limits of transformers have meant that these same principles are often not applicable to genome-based applications, such as CNA calling, where distances between genomic regions of interest might be vast [29]. One of the few examples is ECOLE [30], which is designed for whole exome sequencing (WES), which uses a transformer architecture to classify exome regions into neutral, deletion, duplication, etc. Since WES has lower data resolution as it captures only the coding portion of the genome, ECOLE tackles the simpler task of classifying exome regions into these broad categories and does not call the exact copy number or allele-specific copy numbers.

Deep learning models have been developed in the context of calling germline variants—single nucleotide variants (SNVs) and indels from normal samples [31], as well as some to call SNVs in tumour samples [32, 33]. However, the detection of CNAs from tumour samples is substantially more complex. Unlike germline contexts with a well-defined diploid background, tumour samples have variable purity, with genomes containing many different aneuploid states, obfuscating the true copy numbers. Germline and somatic SNV calling typically focus on local sequence context, while accurate CNA detection requires both global reasoning to infer sample purity and ploidy, and local reasoning to infer changes in copy number states.

In this paper, we propose a novel deep learning-based approach to the CNA calling problem, which we call araCNA, and is based on the use of recently developed transformer alternatives that provide genome-scale modelling capabilities. While existing copy number callers can be considered to be complex signal segmentation algorithms, araCNA learns to call CNAs and other features of sequencing data obtained from cancer samples. We describe how to train such a model and provide empirical evidence of its performance. The approach we suggest therefore opens up new opportunities for creating CNA callers that learn and can be fine-tuned for specific sub-tasks of interest or integrated directly with deep learning models for other data modalities.

## Materials and methods

### Model overview

The input data for araCNA is assumed to be a sequence of allele-specific read counts at genome-wide loci (see Fig. 1A), which can be converted into total read depth (*R*) and BAF (*B*) values as is commonplace for copy number callers. These are fed into a long-range sequence model that converts the input sequence into an output sequence consisting of the probabilities of major and minor copy number values at corresponding loci. Global parameters of interest such as tumour purity or ploidy can also be provided (Fig. 1B).

### Mathematical preliminaries

We first define the mathematical construction, similar to that first shown in [12]. We define *C*_*T*,*i*_ as the total copy number at locus *i* in the tumour, *C*_*P*,*i*_ as the copy number of the paternal chromosome at locus *i*, *C*_*M*,*i*_ as the copy number of the maternal chromosome at locus *i*, and *C*_*B*,*i*_ as the B allele copy number. We further define *ρ* as the purity of the tumour sample (the proportion of tumour versus non-tumour) and *r*_*d*_ as the expected number of sequencing reads per copy number. In practice, samples are sequenced up to a given average read depth, which assumes a uniform coverage of the short reads across the whole genome [34]. When many duplicated regions exist, the actual average read depth per copy number *r*_*d*_ is unknown. Finally, we assign *R*_*i*_ to mean the total number of reads at a locus, and *B*_*i*_ as the BAF. The sequence data is therefore a collection $\lbrace R_i, B_i\rbrace _{i=1}^L$ for *L* loci.

For a pure tumour sample, we have the total copy number as *C*_*T*,*i*_ = *C*_*P*,*i*_ + *C*_*M*,*i*_. Considering sample impurity, we define the sample copy number $C^s_T$ as $C^s_{T,i} = \rho C_{T,i} + 2(1 - \rho ),$ where we assume the contaminating normal cells have copy number 2 at all loci. While normal cells may possess some copy number variants, the size of these regions is typically negligible compared to the cancer-associated alterations we aim to detect, and so we ignore these for simplicity.

The B allele copy number is defined as *C*_*B*,*i*_ = *s*_*p*,*i*_*C*_*P*,*i*_ + *s*_*m*,*i*_*C*_*M*,*i*_, where (*s*_*p*,*i*_, *s*_*m*,*i*_) ∈ {(0, 0), (0, 1), (1, 0), (1, 1)} denotes whether the paternal and maternal chromosomes, respectively, have the specified SNP B allele at locus *i*. Adding sample impurity, we have $C^s_{B,i} = s_{p, i}((1 - \rho ) + \rho C_{P,i}) + s_{m, i} ((1 - \rho ) + \rho C_{M,i}) .$ The total number of reads at a locus *R*_*i*_ are then $R_{i} = r_d C^s_{T,i} .$ While the BAF *B*_*i*_ at locus *i* is given by $B_i = {C^s_{B,i}}/{C^s_{T,i}}.$

Both *R*_*i*_ and *B*_*i*_ are measured data obtained from sequencing a tumour sample. Figure 2 illustrates the relationship from (*ρ*, *r*_*d*_, {*C*_*P*,*i*_, *C*_*M*,*i*_}) → {*R*_*i*_, *B*_*i*_}. Our aim is to do the reverse—to infer (*ρ*, *r*_*d*_, {*C*_*P*,*i*_, *C*_*M*,*i*_}) from {*R*_*i*_, *B*_*i*_}. However, we can see in the above formulation that both *R*_*i*_ and *B*_*i*_ remain the same if we were to swap the values for *C*_*P*,*i*_ and *C*_*M*,*i*_, meaning that we are unable to infer the parental copy numbers—they are non-identifiable. We can instead introduce *A*_*M*,*i*_, *A*_*m*,*i*_ = max (*C*_*P*,*i*_, *C*_*M*,*i*_), min (*C*_*P*,*i*_, *C*_*M*,*i*_) as the major/minor allele-specific copy numbers, which are identifiable. Hence, we amend our aim to infer (*ρ*, *r*_*d*_, {*A*_*M*,*i*_, *A*_*m*,*i*_}) from {*R*_*i*_, *B*_*i*_}.

### The araCNA model

The function of araCNA can be summarized as $f_\theta (\lbrace R_i, B_i\rbrace ) \rightarrow (\lbrace p_{i,k}\rbrace , \hat{\rho }),$ where *f*_*θ*_ is the long-range sequence model parameterized by network weights *θ*. $\hat{\rho }$ is araCNA’s global purity estimate, while *p*_*k*,*i*_ is the probability that araCNA assigns the locus as belonging to copy number profile *K*_*j*_. The profile categories *K*_1_, …, *K*_*J*_ correspond to major/minor parental copy number combinations, with *K*_1_ ≔ (*A*_*M*_ = 0, *A*_*m*_ = 0), *K*_2_ ≔ (*A*_*M*_ = 1, *A*_*m*_ = 0), etc. Here, $J = \sum _{i=0}^{T_\text{max}} \min (i + 1, T_\text{max} - i + 1)$, where *T*_max_ is a hyperparameter corresponding to the maximum modelled total parental copy number. From these, we can also estimate $\hat{r}_d = \mu _{\text{robust}}(R)/{\Phi ^s}$, where *μ*_robust_(*R*) is the robust or trimmed mean of the read depth vector and Φ^*s*^ is the expected value of the overall sample copy number (i.e. sample ploidy).

We trained our model using simulated datasets where the ground truth copy numbers and purity are known. The details of this simulation are discussed later in this section. The loss function consists of a supervised sequence loss and a supervised global loss. The supervised sequence loss is the cross entropy:


\begin{eqnarray*}
{\mathcal {L}}_{ss} = -\frac{1}{L}\sum _{i=1}^{L}\sum _{j=1}^{J} I\lbrace c_i = K_j\rbrace \log (p_{k,i}),
\end{eqnarray*}

where *L* is the sequence length, *c*_*i*_ ∈ *K*_1_, …, *K*_*J*_ is the known target profile of a genomic locus. The supervised global parameter losses are


\begin{eqnarray*}
{\mathcal {L}}_{sr} &=& | r_d - \hat{r}_d |,\nonumber \\ {\mathcal {L}}_{s\rho } &=& | \rho - \hat{\rho } | .
\end{eqnarray*}

The total loss is then given by


\begin{eqnarray*}
{\mathcal {L}} = {\mathcal {L}}_{ss} + \lambda _r {\mathcal {L}}_{sr} + \lambda _\rho {\mathcal {L}}_{s\rho } .
\end{eqnarray*}

We found *λ*_*r*_ = *λ*_*ρ*_ = 1 to work well. The model was trained using a curriculum learning approach, iteratively increasing the simulation complexity.

#### Model architecture

Sequence inputs are entered into araCNA together with global placeholders and projected these through an encoder block, backbone, and decoder block to give the copy number and purity outputs (Fig. 1B and C). Elements used as global estimates (i.e. purity) in the output are appended to the end of the normal sequence, with zero values for read depth and minor allele frequency.

To differentiate these elements from normal elements, we included a global token feature, encoded as 0 for normal elements and 1 for global prediction sequence elements. The normal observational data (read depth and minor allele frequency) are projected into a dimension *d* using a multi-layer perception (MLP) with ReLU non-linearities, while the token feature is projected into dimension *d* using a simple embedding. The two inputs are combined in this projected space to serve as the encoder block for the main backbone of the model. The output from the main backbone diverges into two streams in the decoder—one for prediction of the copy numbers and one for prediction of the global parameters. Both streams use MLPs with ReLU activation, with a softmax to project the copy number stream into a probability over the *J* possible classes, and a sigmoid to project the purity estimate between 0 and 1.

We created two implementations of araCNA, which use two recently developed long-range sequence models, Hyena [35] and Mamba [36, 37], as part of their architecture. These long-range sequence models known as state space-like models (SSMs) were developed to achieve similar results to transformers but avoid using the computationally expensive self-attention mechanism, where the number of operations scales quadratically with sequence length and therefore is prohibitive for genome-scale data [29]. In contrast, Mamba- and Hyena-based models offer training times that scale linearly with sequence length. We call the araCNA models with Mamba/Hyena blocks araCNA-mamba and araCNA-hyena, respectively. Mamba-2 is the more popular of the SSM models, but being constrained to GPUs with Nvidia A100 architecture (for training and inference). Similar to [38], we adapt the blocks to be bidirectional rather than causal (unidirectional) as used in natural language processing (Fig. 1D). Here, an additional Hyena/Mamba-2 operator processes the reversed sequence, and its reverse projection is combined with the output projection from the Hyena/Mamba-2 operator over the original sequence. Hence, each sequence element has access to every other sequence element.

The hyperparameters of araCNA-mamba and araCNA-hyena are outlined in Supplementary Table S1, resulting in parameter counts of 70k and 12.09M, respectively. Each hyena block contains two positional encodings of length *L*_max _ corresponding to a hyperparameter of the maximum input sequence length. For our models, *L*_max _ was set to 1M; hence, the majority of the parameters, 12M, can be attributed to the positional encodings. We note that 70k parameters for araCNA-mamba are relatively small for a modern neural network, especially given the task at hand is not trivial [39].

### Smoothing

Given the noise in real data, many copy number calling methods employ smoothing or thresholding in some way to reduce the number of CNA segments and reduce false positives. For example, ASCAT has a ‘penalty’ parameter that controls smoothing [12], Battenberg has several ‘gamma’ hyperparameters [13], HMMCopy has ‘e’ and ‘strength’ parameters [10], and CNV Kit uses thresholding to segment and call different copy numbers [15]. We also employ a smoothing technique based on the predicted output probabilities.

Here, we wish to find the ‘best’ joint sequence **S** ∈ {1, …, *J*}*^L^*, given the model output probabilities *p*_*k*,*i*_ that a locus *i* is in copy number state *k*. We can find the optimal sequence by balancing the probabilistic evidence of being in a given state with the penalty *λ*_*t*_ of transitioning to a different state as


\begin{equation*}
\hat{\mathbf {S}} = \arg \min _{\mathbf {S}} \left[ -\sum _{i=1}^L \sum _{k=1}^J I\lbrace S_i = k\rbrace \log p_{k,i}+ \lambda _t \sum _{i=1}^{L-1} I\lbrace S_i \ne S_{i+1}\rbrace \right].
\end{equation*}

For a given *λ*_*t*_, we solve this using a dynamic programming approach similar to the Viterbi algorithm used in HMMs for finding the most likely sequence of hidden states. We found *λ*_*t*_ = 500 to be suitable.

### Synthetic data simulation

We generated synthetic copy number profiles using the following procedures:

- *Sampling the number of segments*: We sample the approximate number of copy number segments $\hat{N}_s$ using a mixture approach; first, we sample a uniform variable *u* such that under a user-defined swap probability *q*_*s*_, the number of segments is sampled uniformly between 1 and *N*. When *u* > *q*_*s*_, a Poisson distribution is used to skew sampling towards smaller total segments. This is to oversample harder cases with fewer segments where it is harder to estimate global parameters such as read depth per copy number and purity.
- *Sampling the segment breakpoints*: This is done by randomly sampling $b_1, \dots ,b_{\hat{N}_s}$ breakpoints from 1, …, *L*, the unique set of these breakpoints defines the segments, and $N_s = |b_1, \dots , b_{\hat{N}_s}|$. We only keep the segments that have a minimum segment length of *L*_min _.
- *Sampling the segment profiles*: We sample *A*_*M*_, *A*_*m*_ of each segment from the possible copy number profiles. We inject logic here to preferentially sample profiles closer in copy number to 1-1 when there are fewer segments. This is due to the identifiability issue. When there are more segments, profiles are sampled more uniformly, but still with a preference for lower copy numbers, to inject an implicit bias towards lower ploidy solutions when the model is unsure.

From a sampled profile, we simulate the sequencing read depth and BAF data. Each of the *L* loci is considered a commonly varying SNP. For both parental alleles, *A*_*M*_, *A*_*m*_, we sample each SNP as binomial with a probability of 0.5, we also sample the purity, *ρ*, uniformly from a range between 0.5 and 1. This gives the sample minor allele copy number $C_{B,i}^s$ and the sample total copy number $C_{T,i}^s$. The read depth per copy number *r*_*d*_ is sampled uniformly between 5 and 70, and together with $C_{T,i}^s$ the overall read depth, *R*_*i*_ is sampled from this mean with additional noise. The BAF *B*_*i*_ is sampled using total reads sampled based on *R*_*i*_ and the subset of B-allele reads using a binomial probability of $C_{B,i}^s/C_{T,i}^s$, with added noise.

**Figure 3.**
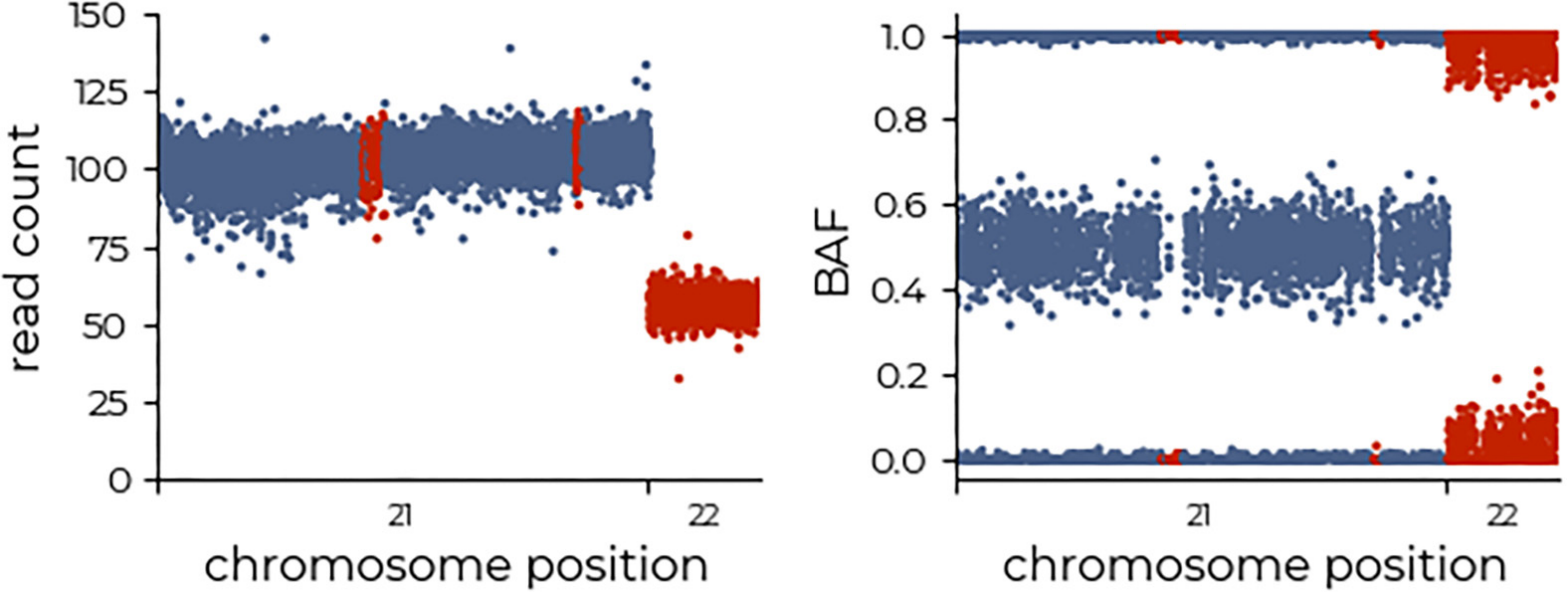
Example of homozygous regions. Real sample example illustrating prolonged homozygous regions on chromosome 21, where the BAF noise is very low and the read-depth is constant. In contrast, although the BAF on chromosome 22 is also around 0 and 1, its increased noise profile and corresponding drop in read depth indicate a deletion with a minor copy number of 0. Red colouring indicates regions of interest.

In real data, there exist regions of prolonged homozygosity that can be attributed to identity-by-descent (IBD) regions (i.e. identical regions inherited from both parents due to a common ancestor; Fig. 3). To emulate this, we also randomly inject regions of prolonged homozygosity into the model. In these IBD regions, the BAF cannot be used to infer copy number, and the model must use context from before/after the homozygous region for correct prediction.

From this sampling procedure, we therefore have a set of targets ({*A*_*M*, *i*_, *A*_*m*, *i*_}, *ρ*, *r*_*d*_) that generate inputs {*R*_*i*_, *B*_*i*_}, which together are used in the training of araCNA. Hence, araCNA can be interpreted as performing inference on the above statistical approach, when ({*A*_*M*, *i*_, *A*_*m*, *i*_}, *ρ*, *r*_*d*_) are treated as unknowns. Further details of this procedure are included in Supplementary data.

### Curriculum learning procedure

For this work, we trained araCNA using simulated copy number profiles (Fig. 1E). We elected to follow this approach since (i) there exists no ground-truth, high-resolution copy number profiles upon which araCNA could be trained, and (ii) we avoid using copy number profiles produced by other methods in order to perform fair comparisons later on. For the simulations, we set the maximum considered total copy number *T*_max_ as a hyperparameter. We use a curriculum learning approach, where the complexity of the simulations is slowly increased and formalized according to:

- Sample the approximate number of segments $\hat{N}_s$ in the total sequence.
- Sample the segment breakpoints that gives the actual number of segments *N*_*s*_ in the total sequence.
- Sample the copy number profiles for each segment, *A*_*M*, *i*_, *A*_*m*, *i*_ (model targets).
- Sample the sequence data *R*_*i*_, *B*_*i*_ (model input) across all segments, based on sampled purity *ρ*, read depth per copy number *r*_d_, and copy numbers *A_M_*_,_*_i_*, *A*_*m*,*i*_.

Noise and tumour purity parameters are sampled randomly during training, allowing araCNA to see different sequence data distributions and learn how to process these into copy number calls. As a consequence, when used at test time for zero-shot inference, it is not necessary to retrain araCNA to process any tumour sample, unlike conventional CNA callers that need to be adapted to each sample every time.

To train araCNA, we adopted an iterative warmup procedure, gradually increasing the complexity of the problem. We found this was necessary for the model to learn, and a similar approach was taken in [40] with gradually increasing the sequence length.

The training procedure was:

- Begin the synthetic data generating procedure with *ρ* = 1, without sampling the noise parameters. Use only up to a maximum total copy number of 2, i.e. profiles (*A*_*M*_, *A*_*m*_) ∈ {(0, 0), (1, 0), (1, 1), (2, 0)}. Sample *r*_*d*_ and start with sequence length 10 000. Train until convergence.
- Using the previously trained model weights as initialization, add in purity and noise parameter sampling. Train until convergence.
- Using the previously trained model weights as initialization, slowly increase the maximum total copy number to 8. Train until convergence.
- Using the previously trained model weights as initialization, slowly increase the maximum sequence length to 650 000. Train until convergence.

We cap the total copy number at 8, as the utility of modelling high-level amplifications (e.g. CN > 8) is limited, often being grouped for downstream analysis, e.g. COSMIC [41] groups ≥9 CNs together. However, a higher total copy number could be easily incorporated with an additional curriculum learning step, increasing training time. Depending on the model’s capacity, increasing the total copy number may also require a larger model, either with increased hidden dimensions or more layers.

### LogR input variant

araCNA was developed initially to require only a tumour sample. However, existing methods often use a normalized read depth input that corrects for mappability and GC bias and usually requires a matched normal sample. To highlight both how araCNA can be fine-tuned with different input data, as well as evaluate araCNA on this more standard data, we also implement an araCNA-logR variant and include its performance on the TCGA data.

This variant takes the araCNA-mamba model, and fine-tunes it using a new simulation procedure. Here, the matched normal read depth $r_d^n$ is also sampled, and instead of *R*_*i*_ as input, the log normalized read depth *l*_*r*, *i*_ is used instead, given by


\begin{eqnarray*}
t_{r,i} &=& \frac{R_i}{2 r_d^n}, \nonumber\\ l_{r,i} &=& \log \frac{t_{r,i}}{\mu _{\text{robust}}(t_r)},
\end{eqnarray*}

where *μ*_robust_(*t*_*r*_) is the robust mean, trimming the top/bottom 5% of data. Further details of the simulation procedure are included in Supplementary data.

This emulates the approach used in ASCAT for real data. We fine-tune this model using samples drawn from this new simulation procedure with initial weights given by araCNA-mamba, training until convergence. In the model, we replace the *R*_*i*_ channel with *l*_*r*,*i*_, and the ${\mathcal {L}}_{\rm {sr}}$ loss term with ${\mathcal {L}}_{s\Phi } = |\Phi ^s - \hat{\Phi }^s|$, where Φ^*s*^ is the sample ploidy, given by $\mu (C^s_T)$. This term links the purity estimate to the output CNs. Given the replacement of *R*_*i*_ with *lr*_,i_, no architecture changes are required; however, if there were (e.g. additional input information), a different encoder model could be used with the backbone and decoder weights given from the pretrained model.

For evaluating araCNA-logR on real TCGA data, we use the output of the ASCAT preprocessing pipeline that corrects for mappability and GC content [12].

### Metrics

We define the metrics used for reconstruction accuracy as follows. The BAF root mean square error (RMSE) is calculated as


\begin{eqnarray*}
&&\sqrt{\frac{1}{L} \sum _{i=1}^L (B_i - \hat{B}_i)^2},\nonumber \\ \hat{B}_i &=& \min _{s_1, s_2} \frac{\hat{C}_{B, i}^s(s_1, s_2)}{\hat{C}_{T,i}^s} \nonumber \\ &=& \min _{s_{1}, s_{2}} \frac{s_1((1 - \hat{\rho }) + \hat{\rho } \hat{A}_{M,i}) + s_2 ((1 - \hat{\rho }) + \hat{\rho } \hat{A}_{m,i})}{2(1 - \hat{\rho }) + \hat{\rho } (\hat{A}_{M,i} + \hat{A}_{m,i})},
\end{eqnarray*}

where the reconstructed BAF $\hat{B}_i$ is calculated from the most probable haplotype (*s*_1_, *s*_2_) ∈ {(0, 0), (0, 1), (1, 0), (1, 1)} at each loci.

The read depth mean absolute error (MAE) is calculated as


\begin{eqnarray*}
&& \frac{1}{L} \sum _{i=1}^L | R_i - \hat{R_i} |,\nonumber \\ \hat{R_i} &=& \hat{r}_d \hat{C}_{T,i}^s, \nonumber\\ \hat{r}_d &=& \frac{\mu _{\text{robust}}(R)}{\frac{1}{L} \sum _{i=1}^L \hat{C}_{T,i}^s}.
\end{eqnarray*}

We opted to use absolute error here, due to mapping errors that can lead to read depth and hence RMSE inflation. Here, *μ*_robust_(*R*) is the robust or trimmed mean of the data, excluding data at the highest/lowest 5% of values.

Battenberg may assign a fraction of each segment to a subclone with a different set of copy numbers [13]. Hence, for the Battenberg multiclonal reconstruction, we account for the fraction attributed to some subclone for that segment. This gives an extra set of parameters for each loci/segment: *τ*_*i*_, the fraction of the sample at that segment attributed to clone 1, $A_{M,i}^1, A_{m,i}^1$, as well as (1 − τ_*i*_), $A_{M,i}^2, A_{m,i}^2$ for clone B. We therefore adapt the reconstruction to follow the Battenberg formulation [13] and in their implemented code (https://github.com/Wedge-lab/battenberg/), where the sample copy numbers for each locus are given by


\begin{eqnarray*}
C^s_{B, i} &=& s_1((1 - {\rho }) + {\rho } (\tau _i A^1_{M,i} + (1-\tau _i)A^2_{M,i}) \nonumber \\ && + \,s_2 ((1 - {\rho }) + (\tau _i A^1_{m,i} + (1-\tau _i)A^2_{m,i}), \nonumber \\ C^s_{T, i} &=& 2(1 - {\rho }) + {\rho } (\tau _{i} ({A}^1_{M,i} + {A}^1_{m,i}) + (1-\tau _i)({A}^2_{M,i} + {A}^2_{m,i})).
\end{eqnarray*}

The analysis for the non-multiclonal Battenberg reconstruction uses the copy number predictions of the clone estimated as having the highest proportion [i.e. max (τ_*i*_, 1 − τ_*i*_)] at each locus, with the normal formulation.

The concordance *CC* between two methods, 1 and 2, is calculated according to


\begin{eqnarray*}
CC_\text{major} &=& \frac{1}{L} \sum _{i}^L I(A_{M, i}^1 = A_{M, i}^2), \nonumber\\ CC_\text{minor} &=& \frac{1}{L} \sum _{i}^L I(A_{m, i}^1 = A_{m, i}^2), \nonumber\\ CC_\text{both} &=& \frac{1}{L} \sum _{i}^L I(A_{M, i}^1 = A_{M, i}^2 \text{ and } A_{m, i}^1 = A_{m, i}^2), \nonumber\\ CC_\text{total} &=& \frac{1}{L} \sum _{i}^L I(A_{M, i}^1 + A_{m, i}^1 = A_{M, i}^2 + A_{m, i}^2) \nonumber\\ &=& \frac{1}{L} \sum _{i}^L I(C_{T, i}^1 = C_{T, i}^2).
\end{eqnarray*}

For methods like HMMCopy, only *C*_*T*_ is output, so only *CC*_total_ can be measured. When method 1 is the ground truth, like in simulations, this can be interpreted as an accuracy.

## Results

### Simulation study

**Figure 4.**
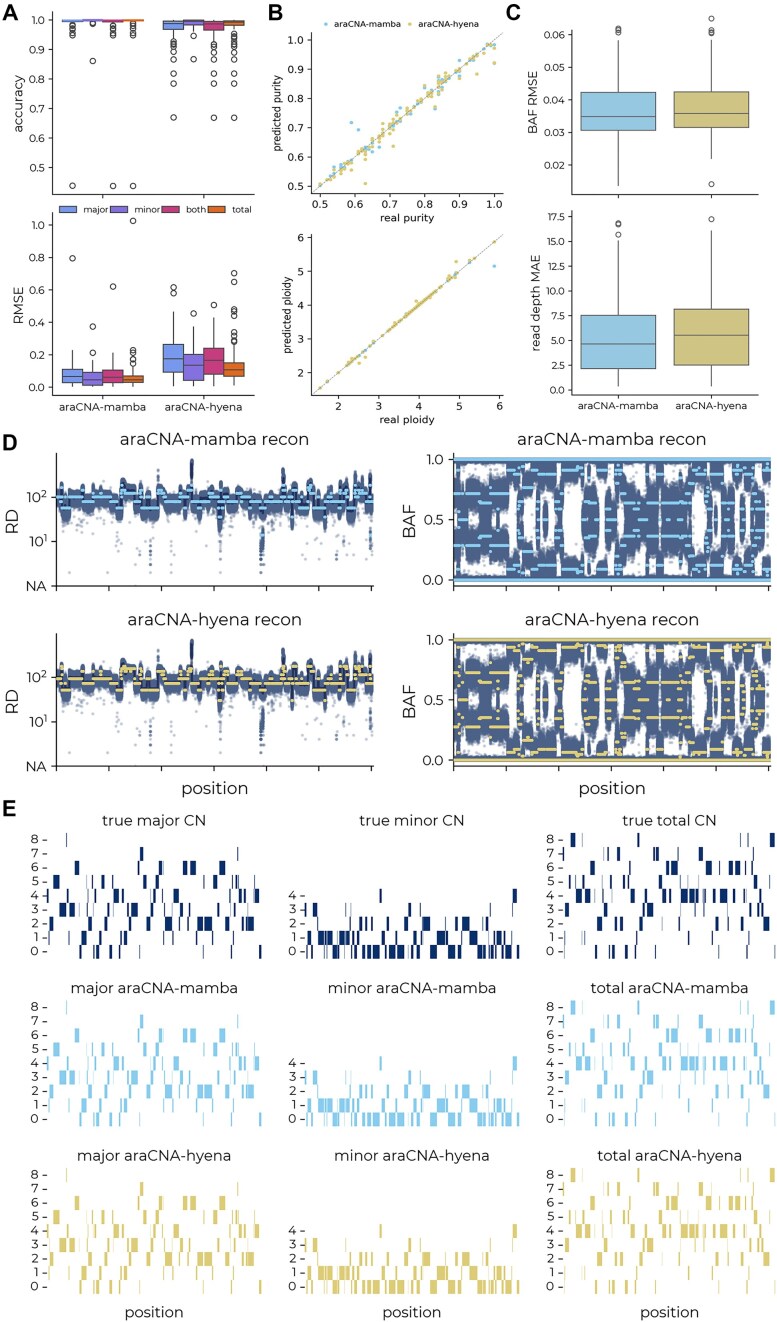
Results for araCNA model variants on simulated data. Panels (A)–(C) show aggregated results across 100 simulated test genomes. (**A**) The distribution of concordance and RMSE between the predicted and true copy numbers. (**B**) The predicted purity and ploidy against the true purity and ploidy. (**C**) The distribution of mean reconstruction error: BAF RMSE and read-depth MAE. (**D**) The reconstructed read depth and BAF and (**E**) the underlying predicted copy number segments for araCNA-mamba and araCNA-hyena on an example simulated test set, the maximum total CN is 8, hence the maximum minor CN is 4 (4, 4).

We first compared results from our two araCNA variants (araCNA-mamba and araCNA-hyena) using simulated data sampled from the same generating procedure from which the training data was also sampled. We show results from 100 simulated test genomes, with a maximum sampled sequence length of 650k, to emulate the data size of a real SNP dataset. Figure 4A shows both models achieve high copy number classification accuracy for the task, though araCNA-mamba slightly outperforms araCNA-hyena. Both models perform well at predicting simulated purity and ploidy (Fig. 4B). Since in this case, we know the ground truth, we can directly measure accuracy. However, in real data, there is no known ground truth, so we also include the reconstruction error as a performance metric (Fig. 4C), where better methods are likely to have a lower BAF and read depth reconstruction. The differences are small, though araCNA-mamba does achieve slightly better metrics than araCNA-hyena. Figure 4D and E show the reconstructed read depth and BAF and predicted copy numbers from each model for one of the example simulated genomes.

The Hyena and Mamba models were trained using the same underlying simulation and training procedure. Differences in results can therefore be traced to differences in the model architecture or sensitivity to hyperparameters. We found that Hyena often took longer to converge during training, becoming stuck in local minima, likely due to the size of the model (12M parameters). In contrast, the Mamba model (70k parameters) achieved better performance despite its significantly smaller size and need for an A100 GPU.

### The Cancer Genome Atlas

We evaluated araCNA on whole-genome sequencing data from a selection of 50 tumour samples chosen from the colorectal (CRC), breast (BRCA), and ovarian (OV) cancer cohorts of The Cancer Genome Atlas (TCGA). We chose these three cancer types due to the extensive and well-characterized aneuploidy present in tumours of these types.

As an initial sanity check, we show that both araCNA-mamba and araCNA-hyena recover diploid states from a normal sample (Supplementary Fig. S1).

We then compared to a number of existing CNA calling tools, two of these are commonly used algorithms that can call allele-specific copy numbers: ASCAT [12] and an adaptation of ASCAT called Battenberg [13], which can also model subclonal populations by optionally assigning a fraction of each CNA segment to another subclone. We compared results to Battenberg without and with its default subclonality modelling.

We also compared to CNV Kit [15], which works with WGS although being intended primarily for WES and gives allele-specific copy numbers after first calling total copy numbers. Finally, we report results for HMMCopy, which only calls total copy number [10]. Both ASCAT and CNV Kit performed well in a recent benchmark [42], while Battenberg and HMMCopy are popular methods included in other method benchmarks [23, 43]. Details of their implementation can be found in the Supplementary data, particularly the justification of the approach CNVkit* (Supplementary Fig. S4).

**Figure 5.**
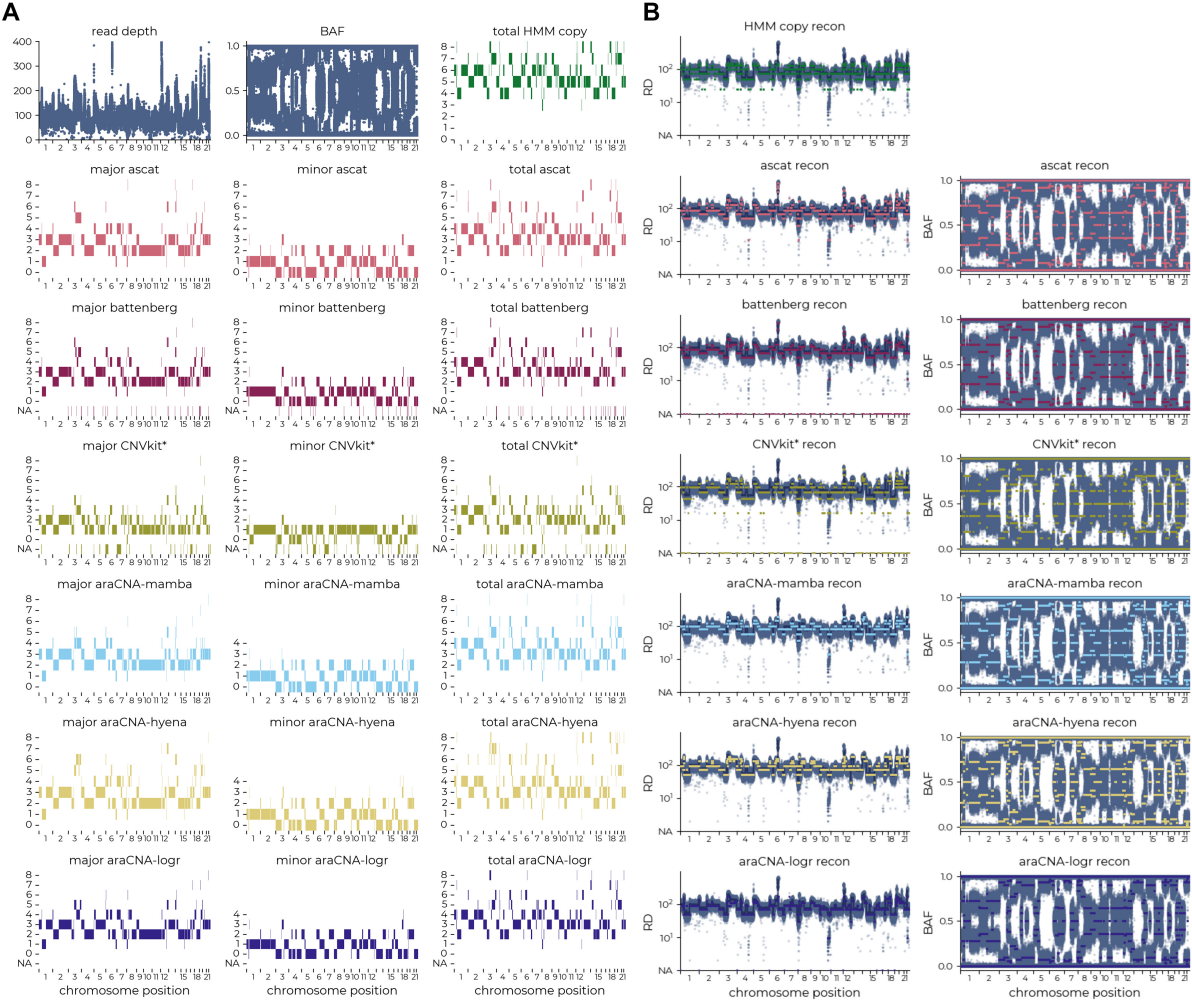
Representative TCGA ovarian cancer sample. Each row shows (**A**) the called copy numbers and (**B**) respective reconstruction of each caller, where araCNA has ∼80% concordance with ASCAT, Battenberg, and HMM Copy.

Since there are no ground truth copy number profiles for these tumours, we used proxy measures such as reconstruction error as in the simulation study to provide an unsupervised metric for performance, where better methods will be expected to have low reconstruction error. However, it is also important to consider both the number of segments and the range of modelled copy numbers produced by each CNA calling approach. A low reconstruction error accompanied by a large number of highly variable copy number segments may suggest overfitting, while high copy number calls at high read depth regions will have a lower reconstruction error but could be less plausible. Figure 5A and B illustrates the analysis of a TCGA ovarian cancer sample using all methods.

**Figure 6.**
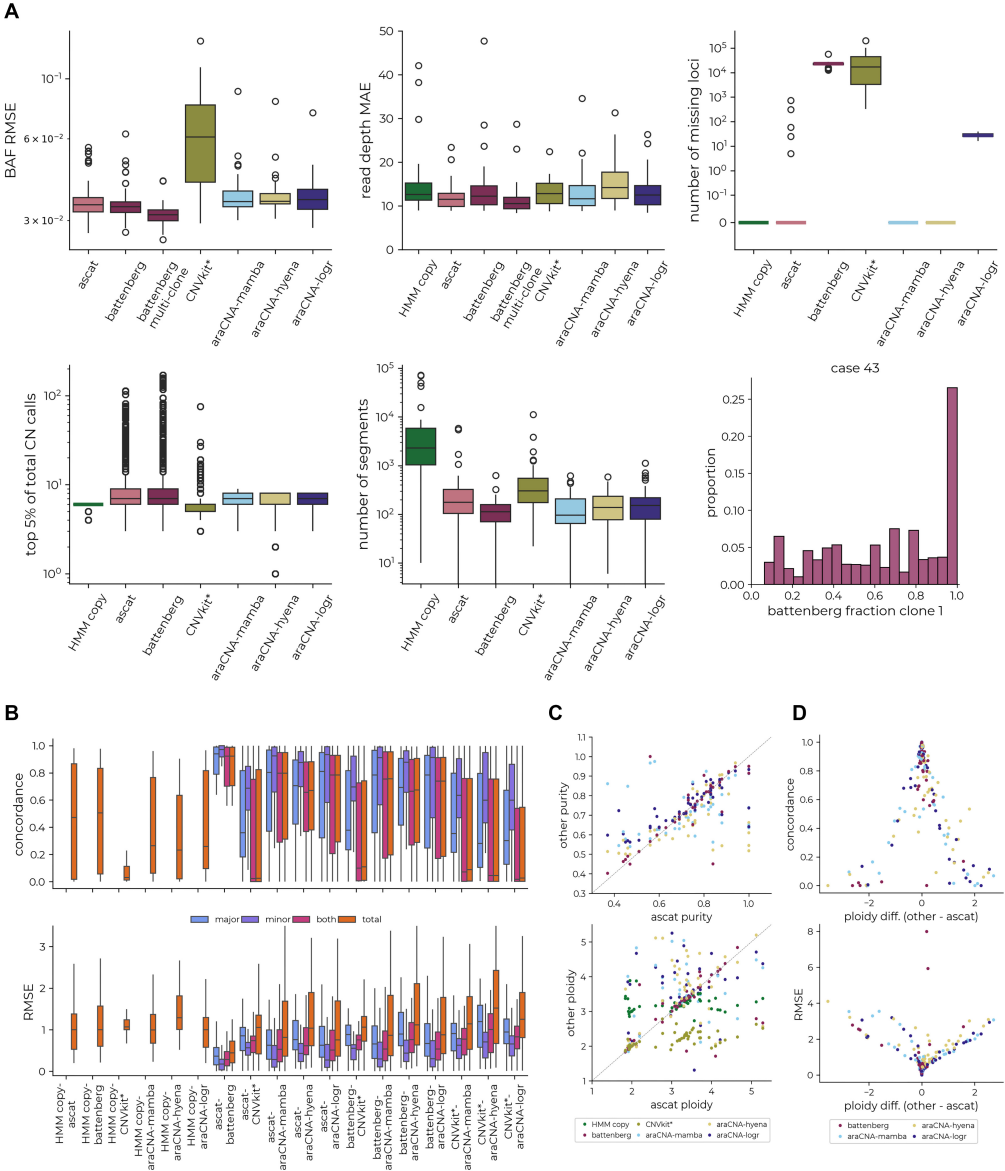
Comparison between CNA callers across 50 TCGA cancer samples. (**A**) Boxplots showing the BAF RMSE, read-depth MAE, number of missing loci after calling, copy number distribution of top 5% of copy number calls, and the number of different copy number segments identified across the tumour samples. Example distribution of Battenberg clonal fraction for a particular tumour. (**B**) The distribution of concordance and RMSE between the copy number predictions of each method. (**C**) The predicted tumour purity and ploidy against the ASCAT-derived purity and ploidy. (**D**) Concordance and RMSE of predicted copy numbers against the ploidy difference between methods and ASCAT.

Figure 6A shows the distribution of the RMSE and MAE for the reconstruction of the BAF and read depth over the 50 samples. Using the three araCNA variants for zero-shot inference of the copy number states gives comparable reconstruction performance to the existing CNA calling methods while using similar numbers of segments. While ASCAT and Battenberg are able to achieve slightly improved reconstruction error performance, when we examined the top 5% of total copy number calls produced, we found that ASCAT and Battenberg sometimes assigned as high as 100 copies to localized genomic regions. It is difficult to verify the correctness of such calls given the lack of ground truth; however, we found most of these regions contained few or no known cancer or other functional genes (Supplementary Fig. S2).

Further, Battenberg’s multi-clonal formulation uses the same input data with more degrees of freedom to model clonal fractions (see the ‘Materials and methods’ section). In the presence of clones, we would expect to see a concentration of fraction values around discrete modes, corresponding to the fraction of the sample originating from a subclone with a diverging copy number pattern across the genome.

We found that in most tumours there were no clear modes in the distribution of clonal fraction estimates (Supplementary Fig. S3), indicating overfitting through this additional degree of freedom, to give the observed lower reconstruction error (Fig. 6A).

We next looked at pairwise copy number classification concordance and the root mean squared difference between copy number calls from different methods (Fig. 6B). araCNA-mamba achieves a median of 80% concordance with ASCAT for all copy number calls across the 50 TCGA cancer samples and a median of 70% concordance with Battenberg, while araCNA-hyena was substantially less concordant with ASCAT and Battenberg. However, when the discrepancies are measured using root mean squared difference, the average differences were much less than one, and we found that most discrepancies were due to small numerical differences (e.g. 1↔2, 2↔3, etc.). When we further examined the differences between ASCAT and araCNA, we found that these appeared to be primarily driven by differences in major and total copy number calls in some samples.

**Figure 7.**
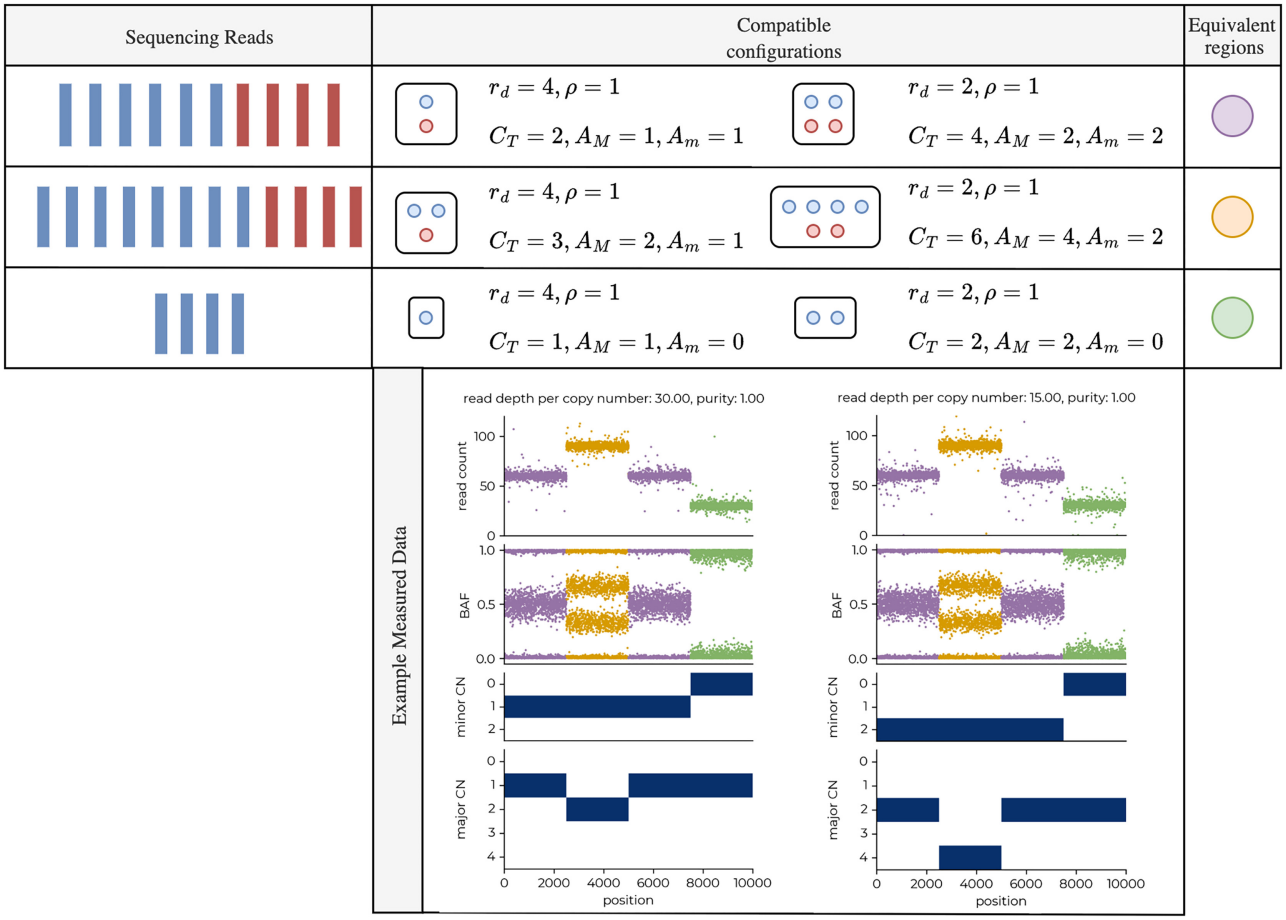
Identifiability of copy number profiles. Example illustrating how different copy number profiles can result in the same observed sequencing read depth and BAF measures. Here, each copy number profile on the right is exactly double that of the left, while the read depth per copy number value is half the left. Blue and red reads are those from the paternal/maternal chromosome, respectively, where the BAF will reflect their chromosome ratio at heterozygous loci. This combination results in the exact same measured data, as highlighted in the bottom panel, where the colours link to each configuration profile above. Parameters are defined in the ‘Materials and methods’ section. In general, many combinations of copy number profile, tumour purity, and read depth per copy number parameters may give rise to similar observed data. In the presence of noise, segments that may uniquely determine the copy number profile can be missed.

To further investigate these discrepancies, we compared tumour ploidy and purity estimates using ASCAT as a baseline. While tumour purity estimates were well correlated between all methods, tumour ploidy estimates differed between methods for some tumours (Fig. 6C). Interestingly, although araCNA is giving a different copy number profile for some samples compared to ASCAT, it still gives low reconstruction error. We surmised that for some tumours there is weak identifiability (see Fig. 7), and araCNA identifies a different ploidy state to ASCAT but with a corresponding copy number profile that is still compatible with the observed data. Indeed, when we examined the concordance between araCNA and Battenberg with ASCAT, we see a reduction when there is a significant departure from the ploidy state estimated by ASCAT (Fig. 6D). Interestingly, where there are disagreements, tumours classified as near-triploid by ASCAT will generally be classified in a higher ploidy state by araCNA and vice versa, which is a hallmark of the identifiability issue. There also exists a subset of tumours where ASCAT and Battenberg disagreed on tumour ploidy despite their algorithmic similarities, which further highlights sensitivity of methods to the identifiability issue.

When considering the effect of using only tumour sample inputs, we see that araCNA-logR, which uses ASCAT-preprocessed matched normal/GC corrected inputs, achieves similar results to the araCNA-mamba model, which uses only tumour read depth. This suggests that preprocessing differences are likely negligible, and the observed variation is more likely due to identifiability issues or high-noise tumour regions that are difficult to resolve.

Overall, while araCNA was trained with simulated data only and applied using zero-shot inference, it appears to perform as well as standard CNA callers on real tumour sequencing data with the caveat that there is a limit to our ability to quantify performance due to a universal lack of ground-truth data. This suggests that this form of simulation-based training is a viable approach to other biological domains, where the underlying generating mechanism is similarly understood, but no real-data ground truth exists. araCNA is faster than other models (Supplementary Fig. S5), performing inference in minutes, as it does not have to fit to each individual sample.

## Discussion

Our results have shown that it is possible to develop a novel deep-learning approach to predict CNAs from whole-genome sequencing. We have built a training simulation framework to generate supervised training data for this biological problem that otherwise would have no known ground truth. We applied our model using zero-shot inference to TCGA cancer samples and found that araCNA achieves comparable performance to existing methods despite being trained only on simulated datasets. This highlights the utility of having strong mechanistic models that relate copy numbers to sequencing data. Overall, these results demonstrate how we can use domain knowledge to develop simulation training sets for simulation-based inference in biological applications, where computing the posterior may be intractable or computationally intensive [44].

We have taken advantage of recent advancements in deep learning, such as SSM, that allow for extremely long sequence lengths, on the order of genomic data, and to learn long-range interactions for fast CNA inference. This approach is not limited to CNA calling and could be repurposed for inference in many biological applications where simulations are already used. Our experiments with Mamba and Hyena highlight that the choice of architecture can be important, and performance can vary between models for a given application.

Importantly, once constructed it is possible to further refine and retrain a model like araCNA with additional data. Unlike classic CNA callers, araCNA can continue to learn and improve using standard deep learning techniques such as fine-tuning or transfer learning. For instance, these approaches may be used to adapt the model due to some distributional shift between the simulated training data used in training and the actual sequencing data. This might occur if, for example, there was additional sequencing noise and artefacts from sequencing of FFPE tumour samples.

While we limited ourselves in this work to training on simulated data, it is possible to use CNA profiles from existing copy number callers for training. However, since these would not be ground-truth data, this would result in a model which is an emulator of the existing copy number caller. While emulators could not normally exceed the original CNA caller in terms of performance, they can offer usability advantages since, by pre-training the emulator, it can be applied directly at run time without retraining on each specific sample. There may also be possibilities for producing ensemble callers by using outputs from multiple existing CNA callers for training.

Our approach could be extended in several ways. Simulations could explicitly model patterns of sequencing variation due to mappability, amplification, or platform sequencing issues. It could also be fine-tuned on real genomes, where profiles have been curated and validated using external data sources (e.g. flow cytometry for ploidy estimation or single-cell whole-genome sequencing). Further, our modelling approach could likely be extended to integrate subclonal calling and holistically account for clone-specific mutations and clone-specific copy numbers. It could likely also be adapted to include multiple longitudinal samples from the same patient, or for low-quality data or different data modalities (e.g. SNP array).

Similarly, araCNA could be adapted to estimate copy number profiles in single-cell data by fine-tuning on low-coverage single-cell simulations. An extension of araCNA could potentially replace the initial cellular CN calling step in pipelines like CNRein [45]. However, summarizing subclonal structure across cellular profiles would require a more complex framework. A similar SSM-based model, taking either raw cellular data or inferred profiles as input to predict representative subclones, could form the basis of future work, particularly given the simulation procedure already introduced in [45].

A challenge we have highlighted is the issue of identifiability when multiple copy number profiles could explain the same measured sequencing data, and the correct copy number profile might be non-identifiable as a result. Multiple tumour sampling in space and/or time, such as used in TracerX [46], can alleviate the problem by increasing the probability of unique solutions but may also introduce issues since different evolutionary trajectories could also produce similar patterns of CNAs. In araCNA, we have addressed this by biasing the training simulations toward lower complexity (ploidy) explanations, as is often done with standard CNA callers. We have found that without such bias, training data in which there exists a one-to-many relationship between input and output causes araCNA to output the average CNA over compatible solutions. Further work is required to enable multiple distinct CNA profiles to be produced.

The way we bias our model towards smaller copy number solutions, when non-identifiable, is through sampling. An alternative would be to introduce an explicit penalty term on total copy number in the loss, discouraging high-CN solutions when non-identifiable. We explored this through attempting to detect non-identifiable sampled profiles and modifying the training target accordingly. However, due to the dependency on purity, read depth, and all segment CNs, detection of identifiability becomes a complex multivariate optimization problem. In practice, it was easier to preferentially sample lower-CN profiles in ambiguous regions. However, a selective penalty in the training loss offers an alternative to changing the sampling distribution that may be simpler or more complex depending on the task.

To clarify the key input requirements and known limitations of araCNA, araCNA can perform copy number inference using a tumour BAM file, without requiring a matched normal, and a SNP file with relevant SNP locations. The araCNA-logR variant can also perform inference using logR/BAF values derived in standard ways with a matched normal and correcting for mappability and GC content, as output by ASCAT. araCNA outputs a CN segment file and a summary information file including the purity, ploidy, and whole-genome duplication estimates. The pretrained models were trained up to the length of our non-problematic SNP list (∼650k), and tolerate some sequence length variation, but may struggle if the input SNP list differs substantially. However, they can easily be fine-tuned up to 1 million SNPs if a higher resolution is required. The better-performing araCNA-mamba pretrained model requires an A100 GPU. Current araCNA pretrained models do not explicitly account for sex chromosomes and are limited to WGS-derived data, though could be fine-tuned to other data modalities as outlined above.

While deep learning approaches in genomics have previously been limited by a lack of labelled ground truth data and sequence length [29], our model araCNA demonstrates how simulation-based learning in tandem with emerging deep-learning architectures can address both these issues. We show how araCNA performs on par with traditional genomic models despite requiring only a tumour sample and not a matched normal sample, fewer markers of overfitting, and performing inference in only a few minutes. On top of this, araCNA has the ability to learn and adapt using fine-tuning or transfer learning.

## Supplementary Material

lqaf124_Supplemental_File

## Data Availability

The latest source codes and tutorials are available on GitHub (https://github.com/e-vissch/araCNA). A permanent archive of the software is also available at Figshare (10.6084/m9.figshare.29063492).
