# Supplementary material for: araCNA: somatic copy number profiling using long-range sequence models
Source: NAR Genom Bioinform. 2025 Sep 9;7(3):lqaf124. doi: 10.1093/nargab/lqaf124 (PMC12418177; doi:10.1093/nargab/lqaf124)
Supplement: lqaf124_Supplemental_File [file lqaf124_supplemental_file.pdf]

Supplementary Information

Hyperparameters

Supplementary Table SI-1 describes the hyperparameters used for the model architectures of `araCNA` and the simulation-based training.

Parameter	Description	Value
**Hyena Backbone Parameters:** unspecified hyperparameters are the same as the defaults provided in Nguyen et al. [1]		
`d_model`	Projection dimension of data after encoder	32
`n_layer`	Number of Hyena blocks	2
`l_max`	Maximum sequence length. Model has capacity for sequences up to this length. Where possible, the model creates arrays of the actual sequence length when less than this to avoid unnecessary memory usage. Some positional encodings of the model cannot be resized and are always set to this value.	1000000
`causal`	Whether to use bidirectional or causal Hyena blocks.	False
`emb_dim`	Dimension of input to inner hyena filter MLP	5
`w`	Frequency of periodic activations of hyena filter	10
**Mamba Backbone Parameters:** unspecified hyperparameters are defaults of Mamba package at commit `7fb78a5`, paralleling their `create_blocks` function and `Mamba2` class.		
`d_model`	Projection dimension of data after encoder and backbone.	32
`n_layer`	Number of bidirectional Mamba2 blocks	2
`expand`	Dimension of A, B, C parameters in SSM, and the factor by which input dimension expands, before projecting back to model dim.	4
`headdim`	Dimension of broadcast projection of the input data, before passing through main Mamba2 block. Can be thought of as implementing `headdim` separate SSMs.	16
`d_intermediate`	Dimension of hidden layer of MLP used as last step in Mamba2 block. If 0, no MLP used.	0
**Encoder/Embedding Parameters:**		
`input_dim`	Dimension of input data (read depth and BAF)	2
`embed_dim`	Dimension of embedding, input dimension into backbone	32
`token_dim`	Number of possible tokens. In `araCNA` only 2 tokens are used- global/not-global, but setting it to 24 allows it to be repurposed in another task with more tokens, e.g chromosome token.	24

Decoder Parameters:

decoder_dim	Output data dimension of backbone, same as backbone input dimension.	32
max_tot_cn	Maximum total copy number that can be modelled, total copy numbers higher than this are mapped to a surplus category.	10

Learning Parameters:

loss_weights	Array corresponding to λ_r, λ_p for the global reconstruction parameters.	[1, 1]
avg_rd_trim_ratio	The proportion of upper/lower quantiles excluded from the average measured read depth in a robust mean calculation.	0.05

Simulation Parameters: these correspond to the final simulation parameters, as a curriculum learning approach iteratively increases the simulation difficulty.

read_depth_range	r_1, r_2	[5, 70]
purity_range	ρ_1, ρ_2	[0.5, 1]
read_depth_scale_range	$\sigma_{r,1}, \sigma_{r,2}$	[0.01, 0.2]
baf_scale_range	$\sigma_{b,1}, \sigma_{b,2}$	[0.02, 0.1]
max_total	The maximum total sampled parental copy number, the sampled minor copy number is less than or equal to the sampled major copy number	8
max_h_segs	$N_{h,\max}$	100
h_l_range	$l_{h,\min}, l_{h,\max}$	[5, 300]
l_min	The minimum segment length, $L_{\min}$.	100
N	The maximum number of sampled segments, N	max(50, L/1000)

Table SI-1: Table of Parameters. Some optional parameters in the code base have not been mentioned as they do not affect the model, and are included for possible later development.

Result on normal sample

As an initial sanity-check to verify `araCNA` calls diploid states in a normal sample, we applied `araCNA` to a randomly selected matched normal from the 50 TCGA sample superset (**Supplementary Figure SI-1**).

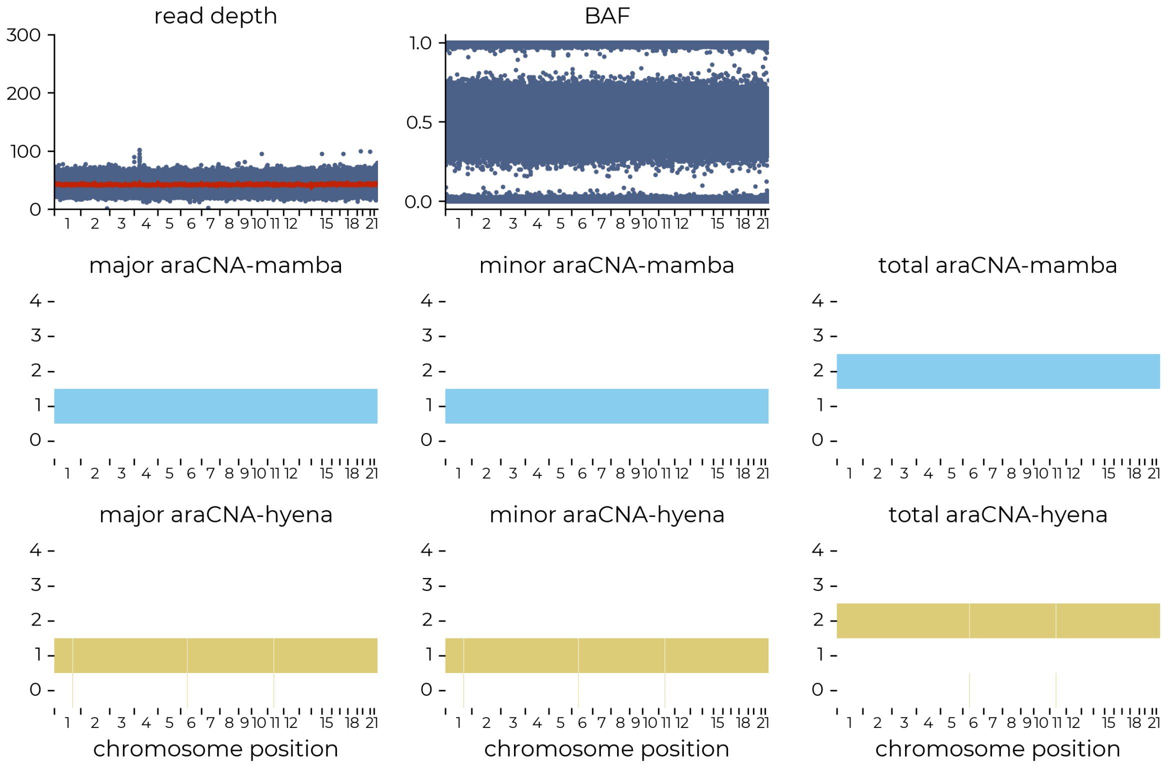

Figure SI-1: Model output on real normal inputs

Caller implementations

The following provides more detailed information about the standard CNA callers used in this study for comparison.

ASCAT

ASCAT uses a piecewise constant fitting algorithm to segment the data based on step changes present in the read depth (it uses the log ratio between normal and tumour), and BAF [2]. It then uses a grid search approach using values for the purity, ρ and ploidy ψ, to find integer copy numbers which best explain the measured data.

ASCAT is implemented according to the to the GitHub, following closely the example given by path `ExampleData/README.md/#ExtractinglogRandBAFfromHTSdataandrunningASCAT`. It requires installation using devtools in R. Please see the araCNA codebase for the exact snakemake workflow used.

ASCAT does not limit the maximum copy number segments are assigned in high read-depth areas, reaching total copy numbers of more than 100 in the TCGA dataset (**Figure 6A**). **Supplementary Figure SI-2** investigates the regions captured by these high CN segments.

Battenberg

Battenberg takes a similar approach to ASCAT, however, may also assign a fraction of each

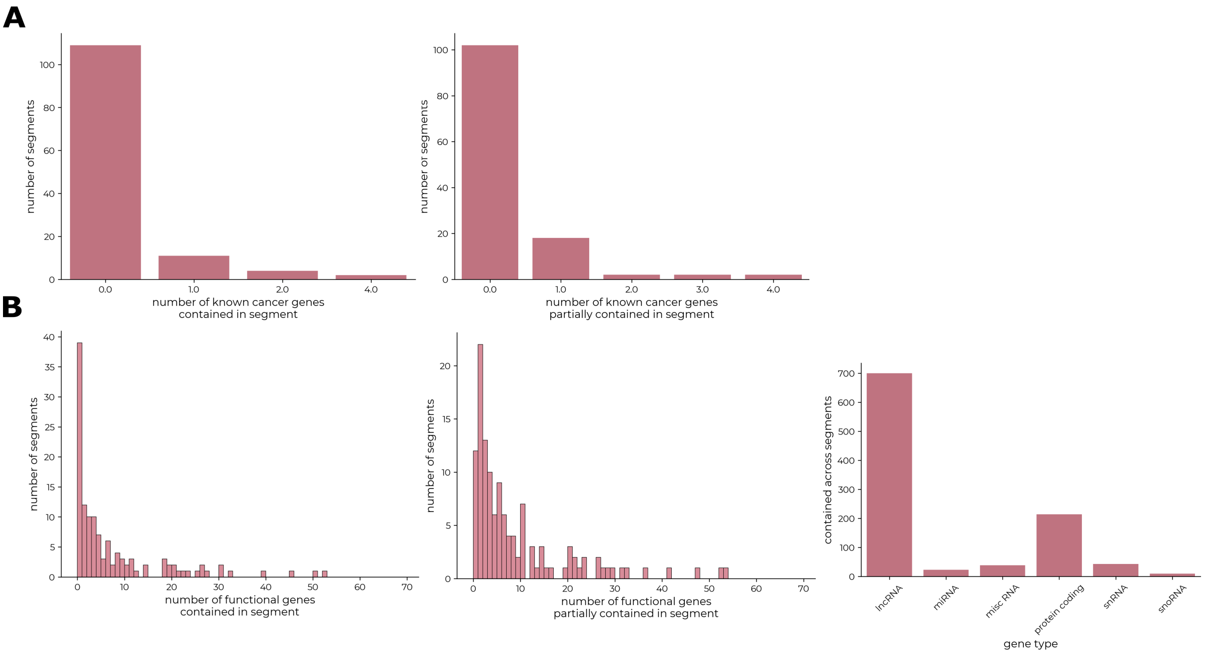

Figure SI-2: Gene coverage of ASCAT focal aberrations. The distribution of (A) cancer and (B) all functional genes wholly or partially overlapped by ASCAT focal aberrations (total copy number ≥ 20). Most ASCAT focal aberrations contain few or no known genes.

segment to a subclone with a different set of copy numbers [3]. This gives an extra set of parameters for each loci/segment; τ_i, the fraction of the sample at that segment attributed to clone 1, $A^1_{M,i}, A^1_{m,i}$, as well as $(1 - \tau_i)$, $A^2_{M,i}, A^2_{m,i}$ for clone B [3]. They also add an additional constraint that $A^2_{M,i} + A^2_{m,i} = A^1_{M,i} + A^1_{m,i} \pm 1$, although multiple copies of a region might occur after the emergence of a subclone. If subclones exist in the data, we would expect the distribution τ_i over segments to be centered around a few distinct modes, where each mode has a different set of copy numbers over regions.

Supplementary Figure SI-3 indicates that in many samples, the Battenberg multiclone solution is likely overfitting to the input data, as there is a somewhat uniform distribution over the fraction of clone 1, when clones are detected. If clones were to exist in the data, we would expect to see a concentration of fraction values around discrete modes, corresponding to the fraction of cells in a subclone where there exists a diverging copy number pattern across the genome. Battenberg may correctly identify clonal population in case 32 and case 40 where it could be argued there are modes around clonal fractions of 0.5. However, for most other samples, it appears that the Battenberg algorithm is likely overfitting to noisy regions by introducing more modelling parameters than is necessary.

Battenberg is implemented according to the GitHub, following closely example given by path `inst/example/battenberg_wgs.R`. It requires the Bioconductor R package 'battenberg'. Please see the araCNA codebase for the exact snakemake workflow used.

HMMCopy

HMMCopy uses only the read depth ratio with normal reads of binned genomic regions, correcting for GC content, and mappability. It uses an HMM to segment the genome into regions of constant copy numbers and call these total copy numbers [4].

HMM Copy is implemented according to the manual, which is available with its installation through Bioconductor. It also requires using HMM Copy Utils GitHub for preprocessing the BAM files. The main script follows the Ontario Institute for Cancer Research GitHub script

run_HMMcopy.R. It should be noted that the HMM parameters sometimes need individual tuning post-inference to obtain fewer total segments and correct the copy number calls [4]. We adopt the same parameter tuning as the script above.

HMMCopy results could perhaps be improved with further parameter tuning on individual datasets, however, we believe that results obtained without manual tuning capture the reduced utility of this tool compared to other methods that do not require such tuning.

Please see the araCNA codebase for the exact snakemake workflow used, including some pre-processing required as described in the HMM Copy Utils GitHub at README.md#Example#0nbinwidths.

CNV Kit

CNV Kit is primarily designed for hybrid capture to infer copy number states, even at regions with very low coverage, however also works for WGS data [5]. It uses circular binary segmentation [6] to segment the genome into areas of constant copy number, then assigns total copy numbers using thresholds on the log read ratio between normal and tumour. After, it uses BAF to estimate the proportion of each total copy number that can be assigned to each allele [7]. Hence, it only uses the BAF to infer allelic copy numbers after initial total copy number calling.

CNV Kit is implemented according to the documentation.

We used the docker image installation, and the 'batch' function pipeline, specifying the method as whole genome sequencing. CNV Kit can also do allele-specific calling if provided with VCFs, so we used VarScan to obtain VCFs of the tumour files at SNP loci, followed by calling with CNV Kit. CNV Kit does not directly account for purity/ploidy but can take both as an input- mainly affecting thresholds for calling copy numbers [7]. Hence, we further implemented CNV Kit with ASCAT purity/ploidy as a final comparison point, as this performed better (**Supplementary Figure SI-4**), we have used this, denoted CNVkit*, as the default in the main text.

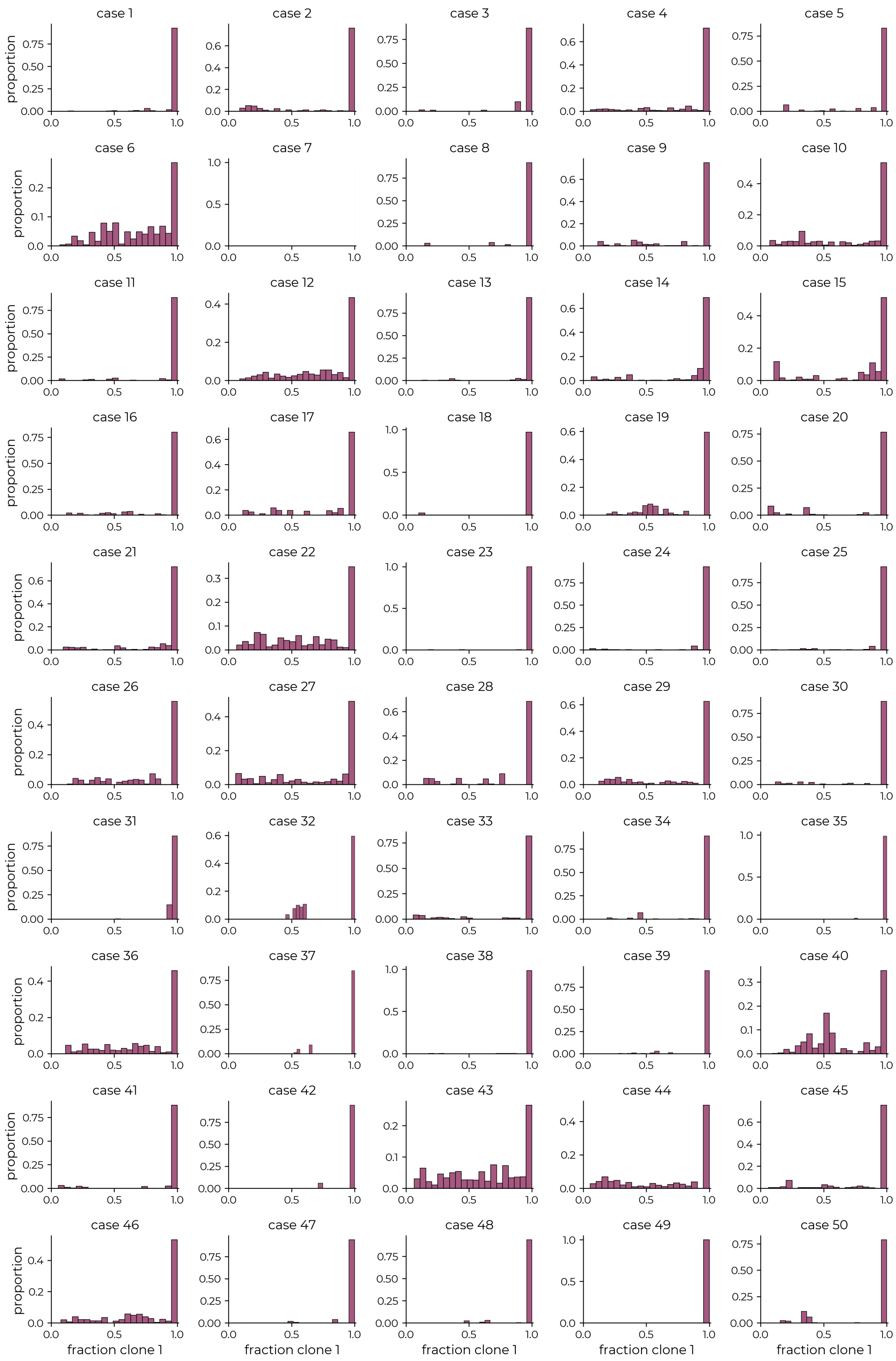

Figure SI-3: Distribution of fraction of sample assigned to clone 1 across loci

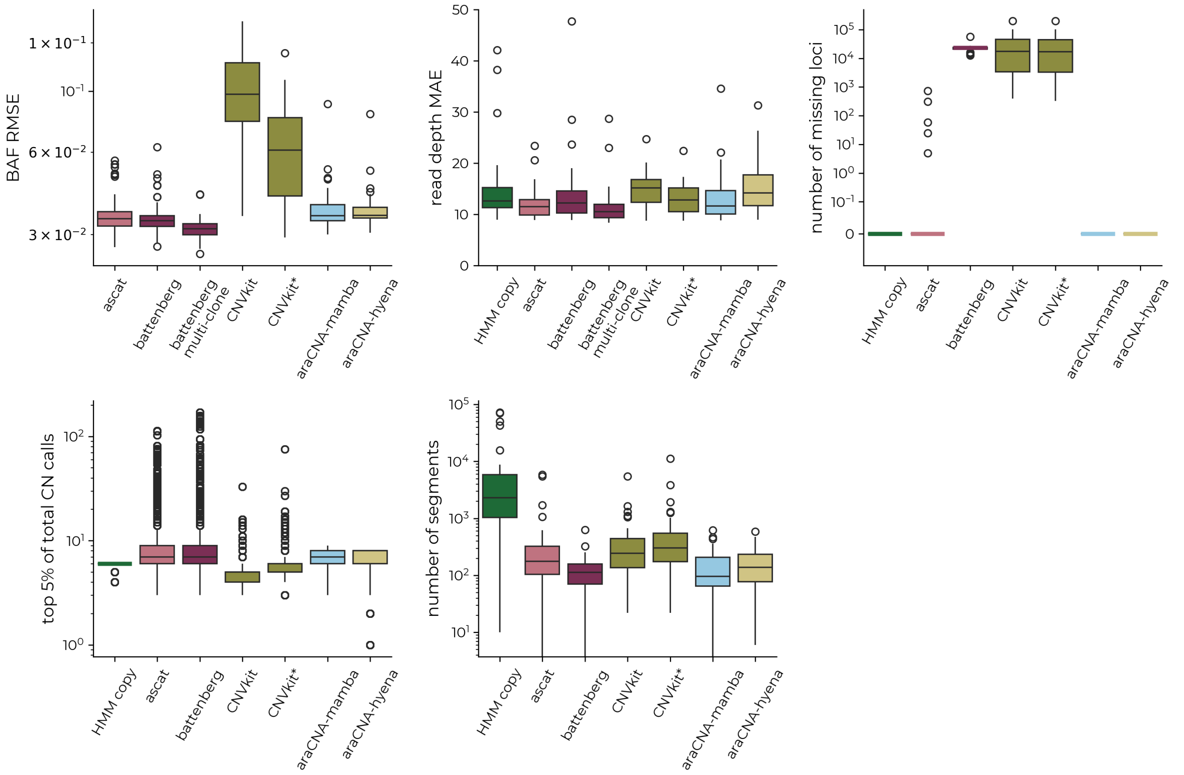

Figure SI-4: Summary metrics on 50 TCGA samples demonstrating that CNV Kit performs better when provided with the approximate purity- taken from the ASCAT estimates.

Additional Simulation Details

To summarise the simulation process mathematically, sets of observation data are simulated based on the sampled parental copy number profiles (A_M, A_m) according to the following scheme:

$$\begin{aligned}
\rho &\sim \mathcal{U}[\rho_1, \rho_2], \\
r_d &\sim \mathcal{U}[r_1, r_2], \\
s_{M,i}, s_{m,i} &\sim \mathrm{Bin}(n=1, p=0.5), \\
R_i &= r_d C^s_{T,i} + r_d \sigma_r \epsilon_{i,1}, \quad \sigma_r \sim \mathcal{U}[\sigma_{r,1}, \sigma_{r,2}], \quad \epsilon_{i,1} \sim \mathcal{T}_2,
\end{aligned}$$

where (ρ_1, ρ_2), (r_1, r_2), $(\sigma_{r,1}, \sigma_{r,2})$ are hyperparameters and $\mathcal{T}_2$ is Student's t-distribution with 2 degree of freedom.

The BAF sampling is slightly more involved to reflect real data observations, and can be described by the following scheme:

$$\begin{aligned}
n_{h_s} &\sim \mathcal{U}\{1, N_{h,\max}\}, \\
l_{h,j} &\sim \mathcal{U}\{l_{h,\min}, l_{h,\max}\}, \quad S_{h,j} \sim \mathcal{U}\{0, L - l_{h,j}\}, \quad j = 1 \dots n_{h_s}, \\
N_{r,i} &= \mathrm{Pois}(R_i), \\
N_{B,i} &= \mathrm{Bin}(N_{r,i}, p = \frac{C^s_{B,i}}{C^s_{T,i}}), \\
\nu_i &= \begin{cases} 1, & \text{if } s_{M,i} \neq s_{m,i} \text{ and } i \notin \cup_j \{S_{h,j}, S_{h,j} + l_{h_j}\}, \\ 0.2, & \text{otherwise}, \end{cases} \\
\sigma_b &\sim \mathcal{U}[\sigma_{b,1}, \sigma_{b,2}], \quad \epsilon_{i,2} \sim \mathcal{T}_{150 * \sigma_b}, \\
B'_i &= \begin{cases} \frac{N_{r,i}}{N_{B,i}} + \nu_i \sigma_b \epsilon_{i,2}, & \text{if } 0 \leq \frac{N_{r,i}}{N_{B,i}} + \nu_i \sigma_b \epsilon_2 \leq 1, \\ \frac{N_{r,i}}{N_{B,i}} - \nu_i \sigma_b \epsilon_{i,2}, & \text{otherwise}, \end{cases} \\
B_i &= \max(\min(1, B'_i), 0),
\end{aligned}$$

where $N_{h,\max}$ is the maximum number of homozygous segments, n_{h_s}, to sample. $l_{h,\min}$ and $l_{h,\max}$ are the minimum and maximum lengths, $l_{h,j}$ of the homozygous segments, and are hyperparameters, as is $(\sigma_{b,1}, \sigma_{b,2})$. We introduce N_r and N_B as the number of normal and B allele reads, to ensure the frequency is centered around rational values, as would be in real data. $S_{h,j}$ denotes the start positions of the homozygous segments, while ν_i is the scaling factor that reduces noise at homozygous loci. The parameter ϵ_2 is sampled from a Student's t-distribution with degrees of freedom, df, that scale with the BAF scale parameter- so that smaller noise samples will have a lower df value to ensure that some extreme values are still sampled.

LogR Simulation Details

For the LogR, l_r simulation procedure, we use a similar procedure to above, but adding:

$$\begin{aligned}
r^n_d &\sim \mathcal{U}[r_1, r_2] \\
t_{r,i} &= \frac{\max(1, R_i)}{2 r^n_d} \\
l_{r,i} &= \log \frac{t_{r,i}}{\mu_{\text{robust}}(t_r)}
\end{aligned}$$

The BAF is sampled as above.

Runtime Comparison

We note that comparing the runtime across models is difficult due to confounding factors:

- Runtime depends on tumour sample complexity
- Some methods require user-managed parallelisation (e.g., by chromosome), while others handle this internally.
- Tumour and normal sample processing can be parallelised differently across methods
- Certain tools (e.g., Battenberg) perform additional steps like phasing, significantly increasing runtime.

Here, we include a runtime comparison on a random selection of 5 TCGA samples. We measure the runtime of our workflow implementations, following documentation procedures for each method as outlined above . For ASCAT, Battenberg, CNV Kit and `araCNA` (preprocessing) we use 24 CPUs. HMM Copy by default does not implement parallelism, so uses only a single core. Inference for both `araCNA` models was evaluated using an a100 GPU, although `araCNA`-hyena can run on a CPU with a slight runtime impairment.

Supplementary Figure SI-5 demonstrates, however, that `araCNA` is faster than other methods, and show that the main bottleneck is the preprocessing (to compute read depth and BAF), which likely could be sped up further, using command line tools rather than pysam (a python library), as we have used.

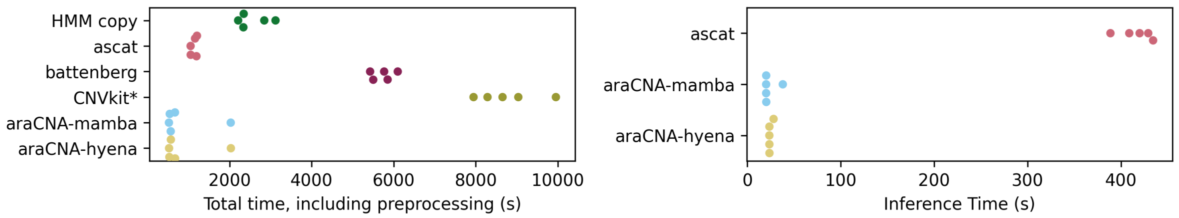

Figure SI-5: Comparison of runtime across models.

Supplementary References

[1] E. Nguyen, M. Poli, M. Faizi, et al. (2023) "HyenaDNA: Long-Range Genomic Sequence Modeling at Single Nucleotide Resolution". *Advances in Neural Information Processing Systems*, 36, pp. 43177–43201.

[2] P. Van Loo, S. H. Nordgard, O. C. Lingjærde, et al. (2010) "Allele-specific copy number analysis of tumors". *Proceedings of the National Academy of Sciences*, 107, pp. 16910–16915.

[3] S. Nik-Zainal, P. Van Loo, D. C. Wedge, et al. (2012) "The Life History of 21 Breast Cancers". *Cell*, 149, pp. 994–1007.

[4] G. Ha, A. Roth, D. Lai, et al. (2012) "Integrative analysis of genome-wide loss of heterozygosity and monoallelic expression at nucleotide resolution reveals disrupted pathways in triple-negative breast cancer". *Genome Research*, 22, pp. 1995–2007.

[5] E. Talevich, A. H. Shain, T. Botton, and B. C. Bastian (2016) "CNVkit: Genome-Wide Copy Number Detection and Visualization from Targeted DNA Sequencing". *PLoS Computational Biology*, 12, e1004873.

[6] E. S. Venkatraman and A. B. Olshen (2007) "A faster circular binary segmentation algorithm for the analysis of array CGH data". *Bioinformatics*, 23, pp. 657–663.

[7] E. Talevich. *CNVkit: Genome-wide copy number from high-throughput sequencing.* 2024.
